# Online Information Exchanges for Parents of Children With a Rare Health Condition: Key Findings From an Online Support Community

**DOI:** 10.2196/jmir.2423

**Published:** 2013-01-22

**Authors:** Florin Oprescu, Shelly Campo, John Lowe, Julie Andsager, Jose A Morcuende

**Affiliations:** ^1^University of the Sunshine CoastFaculty of Science, Health, Education and EngineeringSchool of Health and Sport SciencesMaroochydoreAustralia; ^2^University of IowaIowa City, IAUnited States

**Keywords:** Online social support, Online support communities, Clubfoot, Uncertainty management, Health communication, Health information seeking and provision

## Abstract

**Background:**

The Internet provides new opportunities for parents of children with difficult illnesses and disabilities to find information and support. The Internet is particularly important for caregivers of children with special needs due to numerous health-related decisions they face. For at-risk populations, online support communities can become key settings and channels for health promotion and communication.

**Objective:**

This study is an initial exploration of the information-seeking and information-provision processes present in an online support community, which is an area of opportunity and interest for Internet-based medical research and practice. The aim of this study was to explore and describe information-related processes of uncertainty management in relationship to clubfoot. Specifically, the study explored interpersonal communication (information seeking and provision) in an online support community serving the needs of parents of children with clubfoot.

**Methods:**

The study population consisted of messages posted to an online community by caregivers (parents) of children with clubfoot. The theoretical framework informing the study was the Uncertainty Management Theory (UMT). The study used content analysis to explore and categorize the content of 775 messages.

**Results:**

Women authored 664 of 775 messages (86%) and men authored 47 messages (6%). Caregivers managed uncertainty through information seeking and provision behaviors that were dynamic and multilayered. The ratio of information-seeking messages to information-provision responses was 1 to 4. All five types of information-seeking behaviors proposed by Brashers’ schema were identified, most of them being correlated. Information seeking using direct questions was found to be positively correlated to self-disclosure (*r*=.538), offering of a candidate answer (*r*=.318), and passive information seeking (*r*=.253). Self-disclosure was found to be positively correlated to provision of a candidate answer (r=.324), second-guessing (*r*=.149), and passive information seeking (*r*=.366). Provision of a candidate answer was found to be positively correlated with second-guessing (*r*=.193) and passive information seeking (*r*=.223). Second-guessing was found to be positively correlated to passive information seeking (*r*=.311). All correlations reported above were statistically significant (*P*<0.01). Of the 775 messages analyzed, 255 (33%) identified a medical professional or institution by name. Detailed medical information was provided in 101 (13%) messages, with the main source of information identified being personal experience rather than medical sources.

**Conclusion:**

Online communities can be an effective channel for caregivers, especially women, to seek and offer information required for managing clubfoot-related uncertainty. To enhance communication with parents, health care institutions may need to invest additional resources in user-friendly online information sources and online interactions with caregivers of children with special illnesses such as clubfoot. Furthermore, explorations of information-seeking and information-provision behaviors in online communities can provide valuable data for interdisciplinary health research and practice.

## Introduction

The Internet is particularly important for caregivers of children with special needs due to numerous health-related decisions they face [1]. For at-risk populations, online support communities can become key settings and channels for health promotion and communication [2-4]. However, limited data are available on the uncertainty management behaviors exhibited in online support communities for caregivers of children with clubfoot [5]. In this context, it is important to explore and analyze information seeking and information provision as uncertainty management behaviors [6].

A parent or caregiver experiences uncertainty when caring for a child affected by illness or disability [7-9]. Clubfoot is a developmental disability affecting the lower limb with an incidence of approximately 1 per 1000 live births [10]. Given its visual nature, with one or both feet turned inwards at birth, clubfoot can be a major source of uncertainty for the parents of the children affected after diagnosis and during treatment [5,11]. Uncertainty can also manifest because of the relapses that may occur after treatment [12-14]. Furthermore, available clubfoot treatment options vary and their efficacy depends on the experience of the health care provider [15-17]. Due to the limited time available for consultation with health care professionals and the relative rarity of the condition, caregivers may be likely to turn to online support communities to manage their illness-related uncertainty.

Uncertainty is defined as a psychological state characterized by insecurity and lack of clear information [18]. Similar to other serious health conditions, the parents of children with clubfoot face a complex situation: information about treatment may be unavailable, information about treatment may be inconsistent, and there may be insecurity related to limited general or specialized knowledge about what causes clubfoot and what the long-term effects of the condition are [19,20]. Further sources of uncertainty may include potential stigma of the health condition, child development (including long-term effects of illness), as well as selection and effectiveness of treatment options [21,22]. Faced with such a condition, the parent/caregiver may employ various behaviors to manage uncertainty. However, there are gaps in knowledge regarding uncertainty management behaviors of caregivers, especially in online environments.

### Uncertainty Management Theory

The Uncertainty Management Theory (UMT) addresses a number of ways in which impacted individuals attempt to reduce, maintain, or increase their level of uncertainty [18]. Individuals can manage uncertainty (theirs and others’) through appraisal processes and behavioral responses. Situation appraisals can influence the magnitude of uncertainty and its impact on the individual [23]. Behavioral responses may include information seeking and information provision, the focus of this study. In addition to fulfilling the need for acquiring and sharing information, exchanging information online may help parents feel more secure in their role as a caregiver of a child with clubfoot and validated as members of the community [22,24,25].

### Information Seeking and Uncertainty Management

Information is one of the tools available to manipulate uncertainty [26]. Information seeking may include question asking, self-disclosure, offering a candidate answer, second-guessing, and passive information seeking [18]. However, the distribution of information-seeking behaviors and their correlations is yet to be documented.

In health-related situations, the high stakes may lead to a large number of venues and styles of information seeking to ensure that the individual gains access to as much information as possible regarding a certain health issue [27,28]. The Internet is a venue of increasing importance for health information exchanges. A report from the Pew Internet Research Institute [29] indicates that there has been an explosion of health-related information online, both in terms of production and consumption, because of the increase in Internet access (74% of American adults use the Internet). The Pew survey found that 80% of American Internet users have searched online for health information and that 70% of American Internet users who are also caregivers for an ill person look for health information online. Of those surveyed, 40% of caregivers indicated that online health information had been helpful [29].

### Parents on the Internet

Most clubfoot cases are diagnosed at birth, and some of the new parents may not be able to receive and process sufficient relevant information during postnatal care [30]. As a result, new parents may use the Internet as their major source of health information [31]. It has been documented that most parental Internet users are women [32,33], their mean age is less than 35 years old, and many are first-time parents [34]. However, their patterns of information seeking and provision, especially in online communities are unknown.

Given the increased availability of medical information, more parents want to be part of treatment-related decision making. The increased availability of medical information can enable them to become informed medical consumers [35]. Parents may become dissatisfied with the medical encounter if not given sufficient attention or access to informational resources [36] and leave the consult appointment feeling that they can search for, find, and use medical information that is superior to the recommendations of the physician [35]. To manage uncertainty regarding the health care provider, parents may attempt to identify and recommend the physicians and medical institutions that provide good care while steering away from those who provide less than satisfactory care [37]. Online messages that include identifying information of health care professionals and institutions may be used to manage the uncertainty of the recipient (reduce, increase, or maintain) regarding selection of the medical care provider [38]. Research exploring whether informational exchanges within online support groups mention health care providers and institutions is still in its infancy.

The use of the Internet as an information and support source is of particular importance for parents of children with illnesses [31]. A child affected by illness or disability increases the need for parental support and information [31], and parents who need to care for such a child often become active seekers of information [28]. These parents may attempt to find as much information as possible about the health condition of interest including the best treatments, doctors, and facilities available [37]. Information may be sourced using search engines, websites recommended by friends, advertisements in parental magazines, and online support groups [31,39]. Data describing types of medical information provided in online communication and their origin in the context of clubfoot or similar congenital disabilities are currently unavailable.

### Information Seeking in Online Support Groups

Repeat visits to monitor online groups and obtain updates may be considered as part of the ongoing process of managing uncertainty by having the most up-to-date information and confirming that the information acquired so far is still valid [40]. Online support groups have a number of advantages such as 24/7 availability, lack of geographical barriers, a greater degree of anonymity, and ability for people to carefully read and compose messages [41-43]. Given the relative rarity of the clubfoot condition, another advantage is the ability to find or even meet other parents who face similar challenges in caring for their children with clubfoot. Some disadvantages may include lack of physical contact, potential of negative experiences, and lack of information quality control mechanisms [44,45]. The lack of quality control in online communities, especially when discussing medical issues, may result in information that could be conflicting, misleading, or even invalid [46,47]. Thus, it is important to identify the sources of medical information exchanged in a user-managed online community that is not monitored by health care professionals.

### Study Aim

The aim of this study was to explore and describe information-related processes of uncertainty management in relationship to clubfoot. Specifically, the study explored interpersonal communication (information seeking and provision) in an online support community serving the needs of parents of children with clubfoot. The setting for the study was a user-managed online community for the parents of children with clubfoot. This study adds to the body of research seeking to understand better how parents use online support communities to seek information and to manage uncertainty when caring for children with rare health conditions.

## Methods

### Study Population

The study population was represented by messages posted to the oldest and largest Yahoo-based user-managed online support community dedicated to informational and social support needs of parents of children with clubfoot. In 10 years, the group members exchanged over 76,000 messages. The group had over 2300 members with approximately 20 new members joining each week. Members posted over 50 new messages every week. Active members posted messages to the community, either seeking information or providing information and other types of support.

### Study Sample and Methodology

The study sample consisted of randomly selected messages. The sampling methodology was systematic random sampling [48]. The sampling rate was 100 (every 100th message was collected) starting with a randomly selected message posted in the early days of the group. Researchers collected, coded, and analyzed 775 messages posted between January 2000 and December 2008. The study methodology received ethical approval from the University of Iowa’s Institutional Review Board, and the group owner and administrator provided permission to conduct the research.

The study used content analysis to analyze and code messages. Content analysis is an accepted method to study exchanges in online support communities [42,49,50]. Descriptive variables included gender of author, type of message, and intended recipient of the message. These variables were recorded separately during the data collection and deidentification process. The gender of the author was recorded as female, male, and unknown. The type of message was recorded as original message if it was the first message in the thread or response to a previous message if not the first message in the thread. The intended recipient was recorded as individual if the message was clearly addressed to an individual (ie, addressed by name) or group if the message was not addressed to a specific person.

The types of information-seeking behavior were coded following the schema proposed by Brashers [5]: question-asking, self-disclosure, offering a candidate answer, second-guessing, and passive information seeking.

Messages with identifying information were those that included a clear identification of a facility or medical professional, either by name or by a description. These messages were categorized based on the expressed overall experience of the poster in: negative (“I have to admit I have been left to feel rather neglected through this recent experience with the hospital.”), neutral, or positive (“We could not be happier with our doc. He is outstanding and caring...”).

Messages including information about diagnoses, symptoms, regular, and alternative treatments, relapses, and other medical issues were coded as messages dealing with medical information. The source of medical information was classified as personal if it was based on the author’s experience, medical professional if a health care provider was mentioned as source, hospital or institutional website if a link was provided, and medical textbook or journal if the title was provided.

Statistical analyses, including intercoder reliability, were conducted using the SPSS software package. To compute intercoder reliability, out of the analytical sample, 15% (N=116) of messages were randomly selected and independently coded by 2 coders following the methodology proposed by Neuendorf [48] for intercoder reliability computations. Both coders were trained using 30 messages that were not included in the analytical sample. Both coders maintained coding notes as sources of data for thematic analysis using a naturalistic inquiry approach based on the constant comparison method [51]. During peer debriefing, the coders discussed 14 messages where coding disagreements occurred, and the final coding included the consensus opinion. To ensure validity, disagreements were documented in the coding notes prior to achieving consensus in order to reduce potential biases [49]. Krippendorff’s alpha coefficients were computed for each variable. Krippendorff’s alpha ranged between .84 and .98. A Krippendorff alpha above .80 was considered acceptable [48,52].

##  Results

Out of 775 messages coded, women posted 664 (86%) and men posted 47 (6%) of the messages. The gender of the author was unknown for 64 (8%) of the messages. 620 out of 775 messages (80%) were replies to a previous message, and 155 (20%) were initial messages for a ratio of 4:1, indicating an average number of four responses for each original message posted to the board. The intended recipient was an individual in 559 (73%) of the messages, while in 210 (27%) of the messages the intended recipient was the online support group as a whole.

The most frequent information-seeking behaviors were direct questions in 196 (25.3%) messages, followed by self-disclosure in 116 (15%) messages. Other types of information-seeking behaviors were identified as offering a candidate answer in 33 (4%) messages, passive information seeking in 31 (4%) messages, and second-guessing in 3 (0.4%) messages. Five Pearson product-moment correlations were conducted to determine the correlation between various types of information-seeking behavior. Information seeking using direct questions was found to be positively correlated to self-disclosure, offering of a candidate answer, and passive information seeking. Self-disclosure was found to be positively correlated to provision of a candidate answer, second-guessing, and passive information seeking. Provision of a candidate answer was found to be positively correlated with second-guessing and passive information seeking. Second-guessing was found to be positively correlated to passive information seeking. All correlations reported were significant at the *P*<.01 level (2-tailed); see Table 1 for more detail.

**Table 1:** Correlations between types of information-seeking behaviors.

	Self-disclosure	Candidate answer	Second-guessing	Passive
Direct question	.538 ^a^	.318 ^a^	.059	.253 ^a^
Self-disclosure		.324 ^a^	.149 ^a^	.366 ^a^
Candidate answer			.193 ^a^	.223 ^a^
Second-guessing				.311 ^a^

^a^ Correlation was significant at the .01 level (2-tailed).

Of the 775 messages analyzed, 255 messages (33%) included names of health care professionals or institutions. 84 of the 255 messages (33%) provided detailed comments about the named health care professionals or institutions. Of the 84 detailed comments related to health care professionals and institutions, 54 (64%) were positive. Community members strongly urged the recipient to seek a second opinion or to change the health care provider in 30 of the 84 messages (36%).

Of the 775 messages analyzed, 101 messages (13%) included detailed medical information. Of the 101 messages providing medical information, 45 messages (45%) addressed bracing (special shoes the child wears after the casting is completed), 13 messages (13%) provided general information about clubfoot and the treatment options, 11 messages (11%) addressed relapses, and 11 messages (11%) addressed casting issues. The sources of information identified were personal experience in 60 messages (60%), followed by a medical professional in 20 messages (20%), and medical textbooks/journals or hospitals/institutional websites in 5 messages (5%).

The analysis of coding notes indicated that information-seeking messages were generative, dynamic, multilayered, and repetitive. Information-seeking messages were likely to generate information-provision messages. While an exact count was not possible, most information-seeking messages seem to originate from new mothers who recently joined the community following the clubfoot diagnosis or relapse. Information-seeking messages reflected fear and anxiety. They were dynamic in content with questions ranging from diagnosis, treatment options, treatment problems, bracing problems, and later on relapses. Finally, to elicit information from community members, those in information-seeking mode employed multiple layers of information-seeking behaviors.

A majority of information-provision messages seemed to be authored by parents during or after the treatment. These messages often included a combination of information and emotional support directed to the receiver. During the early years of the community, some members used the content of previous well-written messages to answer new inquiries. During the later years of the community, given the repetitive nature of inquiries, some of the more experienced community members created and maintained a list of “frequently asked questions” and a collection of pictures to help respond more effectively to information requests.

The willingness to identify, generate, and use information needed to manage uncertainty was observed in both information-seeking and information-provision messages. The authors of information-seeking messages seemed to have as a primary objective the management of their own uncertainty. The authors of information-provision messages seemed to have as their primary objective the management of the recipients’ uncertainty.

##  Discussion

The study explored interpersonal communication (information seeking and provision) in an online support community serving the needs of parents of children with clubfoot. The discussion sections include: (1) online group characteristics, (2) information-seeking behaviors, (3) messages identifying health care providers, and (4) messages providing medical information and the sources of medical information. Females posted a large proportion of the messages. The ratio of initial messages to responses was 1:4. All five types of information-seeking behaviors proposed by Brashers’ schema were identified, most of them being correlated. Two thirds of the messages mentioned a medical professional, with one in three of those messages providing detailed comments. One in seven messages included medical information, with the main source of information specified being personal experience.

### Online Group Characteristics

During the study period, the messages posted addressed various issues related to caring for a child with clubfoot. The majority of active members were mothers of children with clubfoot indicating that they are the parent more likely to actively engage in online support groups to find and share information about clubfoot. This supports other literature that suggests mothers take the main responsibility for the health care of the family in general and of the children in particular [37,39,53]. It also suggests that women may be more likely than men to actively use support groups [54]. This finding may inform future health communication initiatives and ensure that messages directed to parents are tailored to the predominantly female audience.

### Information-Seeking Behaviors

The most frequently used types of information-seeking behavior in the online community were direct questions and self-disclosure, both of them linked with attempts to manage uncertainty. Furthermore, community members tended to use combinations of information-seeking behaviors designed to generate responses from the community and to build trust. This may allow them to manage multiple layers of interconnected uncertainties: both knowledge-related and interpersonal-related [20]. Information-seeking behaviors were the starting point for exchanges of information in online communities, creating conditions for combining knowledge from multiple sources such as individuals, health care professionals, and Internet-based resources [55].

### Messages Identifying Health Care Providers

According to UMT, individuals use information and other means to increase, maintain, or decrease their uncertainty levels [5]. The results suggested that the main purpose of the clubfoot online community is to allow caregivers to request and exchange information and other types of support. Much of the information provided seems to originate from the personal experience of caring for a child with clubfoot. Such information, in addition to providing solutions to various issues encountered over the course of the treatment, also included names of health care professionals and accounts of hospital visits. Two thirds of the comments regarding physicians and clubfoot treatment were positive. Message distribution suggests that the purpose of the community was not to serve as a scoreboard for physicians or medical care institutions. However, where necessary, group members will urge for second opinions or even for changing medical care providers if the accounts of medical visits do not fit standards of treatment or if some members of the message board had negative experiences with a particular physician or hospital.

### Medical Information

The fact that hospital and institutional websites were rarely the source of medical information posted in online message boards is a key finding since health care information is one of the top subjects that people are looking for on the Internet, with over 12.5 million searches per day focusing on health issues [35]. Additionally, more and more parents want to have as much information as possible about the health care and condition of their child [27,28].

One major reason for illness-related uncertainty is the lack of clear, accurate, and complete information from a trusted source [20,22]. Hospitals and medical care providers represent a trusted source of medical information [35], and yet it is important to note the limited reliance on and reference to medical information available on the websites of medical institutions. This may have three potential explanations: the information is not available, reliable information is difficult to find, existing information is difficult to understand, or physicians do not encourage their patients to take advantage of the existing reliable Internet-based information. While some hospital websites may provide clear and accurate information, caregivers rarely mentioned this information source.

The quality and effectiveness of medical information on the Internet is an issue of concern for health communication scholars and health care practitioners [31,56]. For enhanced health communication, physicians may need to become familiar with high-quality and reliable information websites. This will allow them to increase the quality of information available to parents and to manage their uncertainty in the context of clubfoot care and other similar health conditions. Therefore, it is suggested that medical institutions dedicate additional resources needed to create high-quality online resources providing information on various aspects of the health condition [57]. The existing medical institutional websites in general are perceived as less attractive and difficult to use [58]. Furthermore, in the context of clubfoot, it is suggested that health professionals recommend reliable websites to their clients during the medical encounter as an Internet prescription [44]. This may increase client satisfaction and efficiently use the available consultation time by answering critical care questions and directing clients to online resources such as online support communities [59].

In this context, both medical encounters and online support communities could be efficient as diffusion mechanisms for medical information. The online environment appears to offer excellent opportunities for health care professionals and health communication professionals to provide high-quality medical information to caregivers who are in information-seeking mode [57]. The online environment also offers opportunities for innovative interdisciplinary research that can use information technology to bridge gaps between nursing, medicine, and health communication among others [43,45,57,60].

###  Study Limitations and Future Research

First, this study was limited to an online support community dedicated to caregivers of children with clubfoot. Thus, generalizations to other conditions may not be possible. Future studies may need to explore and compare information-exchange processes in online communities dedicated to other conditions. Current advances in online content research such as natural language processing may be appropriate for analysis and comparison of large datasets across health conditions and disciplines [60].

Second, it is important to note that the members of any online community differ in their levels of participation [61]. There are members who write messages frequently, members who write occasionally, and members who only read messages. The data collected in this study did not allow for such a categorization of participants. Future research may need to examine online information exchanges while considering participation frequency and length. Furthermore, we do not know information-seeking behaviors for those who did not join the group. It may be that some caregivers do not use this community for various reasons and their information-seeking behaviors may be different [62].

Physician-to-patient interactions are one-to-one interactions, while interactions in the context of online communities are one-to-many and many-to-one. The online environment may allow one-to-many and many-to-one interactions to be relatively easy, efficient, and effective by combining the knowledge and intelligence of the group members and preserving the output in a written and persistent format. This is an advantage of online communities that deserves more attention and that will require further interdisciplinary research, for example, modeling the communication pathways and individual involvement in the online exchanges in accordance with the research pioneered by Bambina [63]. Such research could provide direction for additional ways that health care professionals could become more involved in the fabric and activities of user-managed online communities dedicated to caregivers of children with special health conditions. Finally, future research could employ collaboration between members of online communities and scholars from various disciplines [64,65].

###  Conclusion

There is an increasing interest among health practitioners and scholars in the Internet-based behaviors of parents of ill children because of the need to increase effectiveness of health communication initiatives. This paper described information exchanges in the context of an online support community created and managed by parents of children with clubfoot. Caregivers seeking and providing information in online communities can fill a critical gap through communication processes that are timely and relevant. To enhance communication with parents, health care institutions may need to invest additional resources in user-friendly online information sources and online interactions with caregivers of children with special illnesses such as clubfoot. Furthermore, explorations of information seeking and provision behaviors in online communities can provide valuable data for interdisciplinary health research and practice.
